# Using GIS for spatial analysis of rectal lesions in the human body

**DOI:** 10.1186/1476-072X-6-11

**Published:** 2007-03-15

**Authors:** Jane L Garb, Sabha Ganai, Ric Skinner, Christopher S Boyd, Richard B Wait

**Affiliations:** 1 Baystate Medical Center, Springfield, USA

## Abstract

**Background:**

Geographic Information Systems (GIS) have been used in a wide variety of applications to integrate data and explore the spatial relationship of geographic features. Traditionally this has referred to features on the surface of the earth. However, it is possible to apply GIS in medicine, at the scale of the human body, to visualize and analyze anatomic and clinical features.

In the present study we used GIS to examine the findings of transanal endoscopic microsurgery (TEM), a minimally-invasive procedure to locate and remove both benign and cancerous lesions of the rectum.  Our purpose was to determine whether anatomic features of the human rectum and clinical findings at the time of surgery could be rendered in a GIS and spatially analyzed for their relationship to clinical outcomes.

**Results:**

Maps of rectal topology were developed in two and three dimensions. These maps highlight anatomic features of the rectum and the location of lesions found on TEM.  Spatial analysis demonstrated a significant relationship between anatomic location of the lesion and procedural failure.

**Conclusion:**

This study demonstrates the feasibility of rendering anatomical locations and clinical events in a GIS and its value in clinical research.  This allows the visualization and spatial analysis of clinical and pathologic features, increasing our awareness of the relationship between anatomic features and clinical outcomes as well as enhancing our understanding and management of this disease process.

## Background

*This paper addresses technical considerations in rendering data on transanal endoscopic microsurgical outcomes in a GIS. It is a companion to the clinical paper published in the Journal of Gastrointestinal Surgery *[1].

GIS is a term often interpreted in reference to a system of technology, characterized as Geographic Information Systems, which includes data, computer hardware and software, people, and rules. GIS can also refer to the application of GIS technology to the analysis and presentation of geographic features and their attributes, characterized as Geographic Information Science. GIS also refers to a discipline or career path where both the technology and science meet. Some even use "GIS" as a verb, as in "to do GIS." The application of GIS to human anatomic and pathologic features in both contexts, as a system of technology and as a science, has been infrequent.

The most extensive use of GIS in visualizing anatomic features is by the Biomedical Informatics Research Network (BIRN) project[2]. They used GIS to integrate data on brain anatomy from many different sources and registered them in a common three-dimensional coordinate system. They employed the stereotactic coordinate system used in neurological research, clinical diagnosis and neurosurgery to locate specific areas of the brain in three planes (dorsal/ventral and anterior/posterior, medial/lateral). Another use of GIS in clinical medicine has involved Magnetic Resonance Imaging (MRI), where data in a variety of vector and raster formats are converted into topologically correct polygon coverages in ArcGIS, and then web-enabled in ArcIMS. The end-product has become an online, web-based, digital atlas called "SMART Atlas", which allows for customized visualization and query[3].

Use of GIS in visualizing pathologic features is exemplified through use in mapping of lymphatic drainage patterns in melanoma for over 4000 patients undergoing lymphoscintigraphy[4,5]. The absence of a reference system (i.e. common reference points) with this technique made imaging the location of lesions approximate. The investigators used a grid of the lymphatic system to record lesions originally recorded on a hand-drawn sketch of the body. Lesions were assigned to a grid and randomly allocated to a point within the grid cell[6]. This method is currently used strictly for visualization, not spatial analysis.

Two other groups have used GIS to map anatomic features. The first group utilized GIS for dental charting in humans [7]. The second group applied GIS to mammal anatomy, studying the effects of ionizing radiation on microvascular networks in hamsters[8]. They used GIS to collect and store information on individual vessels, calculate transit time of blood flow along the vessels, and visualize vessels as network features using color maps. All of these studies relied on GIS only for visualization of anatomic features, rather than for spatial statistical analysis.

We present a paper that extends the use of GIS in anatomy to study the relationship of human anatomic and pathologic features as well as clinical outcomes in two and three dimensions. The purpose of this study was to determine whether a model of rectal anatomy could be derived in a GIS and integrated with patient clinical data on rectal pathology and treatment outcomes. Our goal was to associate clinical outcomes with location using positional data determined from patients.

The management of rectal polyps and cancers has been conventionally with approaches requiring major abdominal surgery, often requiring patients to have an ostomy, a connection made between bowel and abdominal wall. We chose to study clinical data examining the performance of Transanal Endoscopic Microsurgery (TEM), an infrequently-performed and challenging minimally-invasive procedure used to remove benign and cancerous lesions from the rectum, allowing patients to potentially avoid major abdominal surgery. Surgeons who perform this technique are limited by constraints related to both the position of the polyp within the rectum and the narrow dimensions of the operative rectoscope. We wanted to identify the spatial, clinical and operative characteristics of lesions associated with procedural failure, which included a peritoneal breach (entry into the abdominal cavity) or the inability of the surgeon to remove the polyp with TEM, requiring conversion to open abdominal surgery. Predictive information on characteristics related to these outcomes could later guide the surgeon to choose an alternative procedure involving abdominal surgery instead of attempting and aborting TEM.

The endoscope used in TEM is a rigid tube 4 cm in diameter and 20 cm in length. We used the dimensions of this endoscope to represent the anatomy of the rectosigmoid as a three-dimensional cylinder, which is a valid assumption in the context of the procedure. The cylindrical shape of the endoscope and a uniform point of insertion serving as a reference point facilitate its representation in a GIS in two or three dimensions. Although in reality the rectum is undulating and irregular, if it were stretched out end-to-end, it would resemble a cylinder.

The location of each lesion excised by the TEM procedure is recorded in radial degrees (clock face position) and distance from insertion of the endoscope in centimeters. These lesion locations can be represented as "events" or points in the GIS, and overlaid on the rectal anatomic "map". By organizing patient clinical data, including lesion location, size, pathology, patient age, gender, and clinical outcome, we were able to perform spatial analysis of predictors of poor outcome utilizing spatial regression analysis. We developed this methodology to test the hypothesis that the location of lesions in the rectum influences the probability of procedural failure of TEM.

## Methods

After institutional review board approval, clinical data on TEM findings, operative details and clinical outcomes data were obtained by retrospective review of 144 patients who underwent TEM at Baystate Medical Center in Springfield, Massachusetts, USA, from November 1993 to October 2004.

A map of rectal topology was first developed in the GIS, highlighting existing anatomic features. In the operating room, the surgeon recorded the location of each lesion found endoscopically in terms of clock face position (translated to radial degrees from 0–360) and distance (cm) from the dentate line, a transition zone between anus and rectum. We describe below how both lesion type and rectal topology were rendered in two and three dimensions in a Geographic Information System (GIS). A cylindrical coordinate system was used to convert data from two to three dimensions, using ArcGIS [9].

### Two-dimensional (2D) rendering

**Figure 1:**
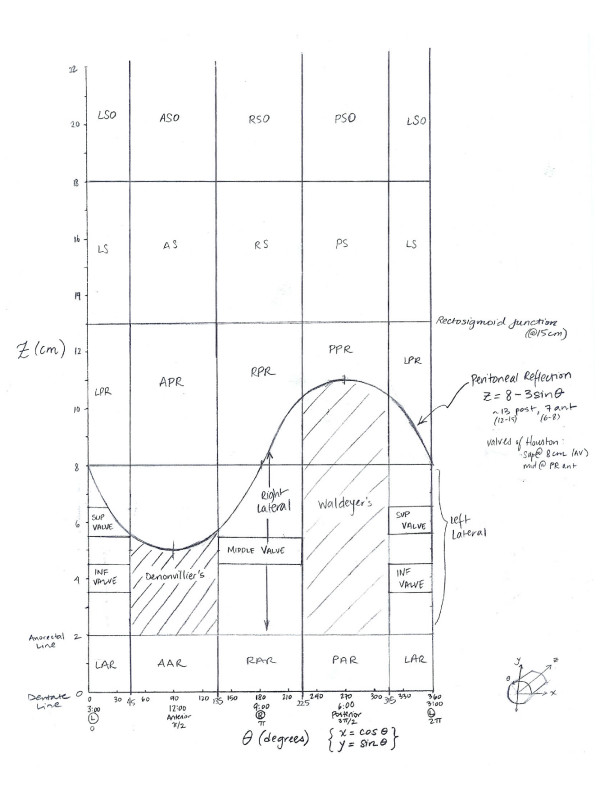
**Sketch of anatomic regions of the rectum**. The X-axis position on the rectal wall on four different scales: radial degrees, clock face position, quadrant, and radians. The Y-axis represents distance from the dentate line in centimeters. Note that the boundary of the peritoneal reflection feature is represented by a sine wave, and boundaries of other common features as well as the limit of the rectoscope are represented by straight lines.

Figure 1 shows a sketch of the proposed rectal anatomy schematic. The rectum is represented in two dimensions using cylindrical projection: a cylinder split lengthwise and spread onto a flat surface. Anatomic features or lesions on the wall of the rectum are plotted on the x-axis on four different scales: radial degrees, centimeters, clock-face position and quadrant. Radial degrees were converted to centimeters by a simple trigonometric function based on the diameter of the scope (This is detailed in Appendix 1). The distance along the rectosigmoid colon from the dentate line was plotted on the y-axis in centimeters.

**Figure 2:**
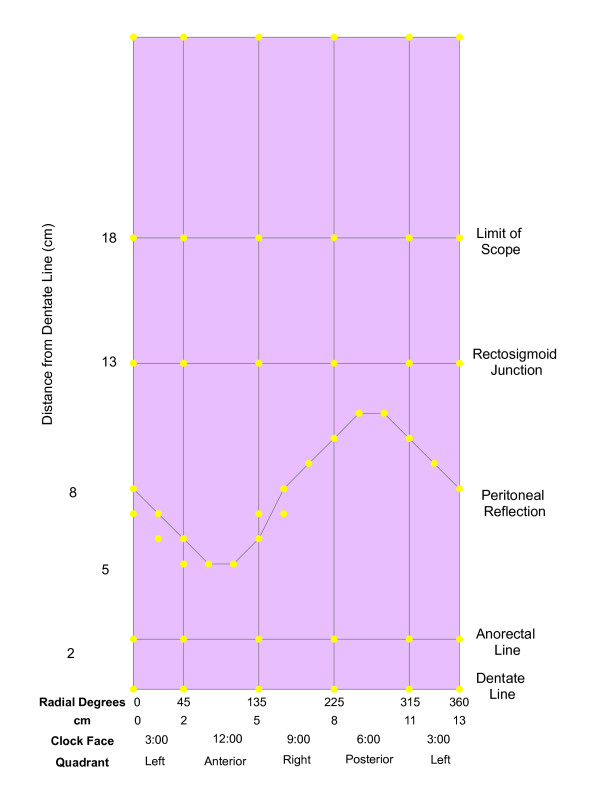
**Bounding coordinates and polygons for anatomic regions**. Distance and position on the rectal wall were entered into the GIS and plotted as points on a two-dimensional plane. The x-axis represents position on the rectal wall, measured in either radial degrees, centimeters, clock-face position, or quadrant. The y-axis represents distance from the dentate line measured in centimeters. Polygons were then created by digitizing from bounding coordinates.

Figure 2 shows the sketch in Figure 1 translated into a map of rectal topology in the GIS, highlighting clinically-relevant anatomic features. The map was defined by discrete equations and boundaries that allowed for a simplified representation of a rather complex human organ, while retaining utility through its ability to logically and categorically partition variables according to contextual relationships.

To construct the map, the rectum was divided into regions (polygons) bordered horizontally by the boundaries of anatomic landmarks and vertically by directional quadrants (Figure 1). Position on the rectal wall and distance from the dentate line for the bounding coordinates of these regions were entered in an attribute table in the GIS and plotted as points (Figure 2; details are provided in Appendix 1). The peritoneal reflection (an obliquely positioned boundary between the abdominal cavity and the pelvis) was plotted as a sine wave, with y-values calculated as a function of position on the rectal wall. Polygons were subsequently created by digitizing these points (Figure 2).

Lesions detected on TEM were rendered as points in the GIS by converting their distance and clock face position to x- and y-values. Attribute data for each lesion consisted of pathologic characteristics (lesion size, histology, presence of positive margins on excision, whether the lesion was a recurrence) and the occurrence of procedural failure, which was defined as peritoneal breach or conversion to open surgery. Using a point-in-polygon operation, the name of each anatomic region was attached to the lesions (points) within it. Attribute data for the lesions were then aggregated over regions so that a summary rate or value on various lesion attributes could be calculated for each region and visualized through choropleth mapping.

### Two-dimensional (2D) visualization and statistical analysis

#### Visualization and exploratory spatial analysis

A dot map was initially used to display lesion location as points. Choropleth mapping was then used to summarize various pathologic characteristics and procedural failure rates for the anatomic region (polygons). Maps were visually inspected to identify patterns and generate hypotheses as to the relationship of region characteristics and procedural failure, to be tested with mathematical modelling. Spatial autocorrelation (clustering) of failure rates across anatomic regions (polygons) was tested using Moran's I-statistic [11].

#### Mathematical modelling

Ordinary least squares regression was used to model clinical and locational factors in procedural failure. The unit of analysis in the regression was anatomic region. Failure rate of each region was the dependent variable. Visual inspection of the choropleth map of regional failure rates and comparison with choropleth maps of other regional pathologic characteristics (for example average lesion size or percentage of lesions with positive margins) suggested the independent variables for the regression.

A global Moran's I-statistic [10] was then applied to the regression residuals to test for spatial autocorrelation (clustering). A significant finding of this statistic confirms the presence of spatial variation in the data which is not explained by the regression model and indicates the appropriateness of fitting a model with spatial dependence. Spatial regression models large-scale variations in the dependent variable due to spatial location of the regions and other covariates and small-scale variation due to interactions with neighbors. In this analysis, generalized least squares regression was used with a first-order (rook) neighbourhood contiguity matrix and a conditional autoregressive spatial covariance structure[11]. The S-Plus extension for ArcView GIS [12] was used for all analyses.

### Three-dimensional (3D) rendering

In three dimensions, the rectum was represented as a cylinder, and anatomic regions represented as partitions of the cylinder. Each partition's bounding cylindrical coordinates as derived in 2D (θ, d) were converted to Cartesian coordinates (xyz) using trigonometric functions (Appendix 1). The negative of the distance from the dentate line (the y-coordinate in 2D) represented the z-factor. The result became a series of overlapping points at different heights (z-values of 0, 2, 13, 18 and 26 cm) in a circular pattern.

A line feature class was then created by joining sets of points every 5 degrees around the circumference. Line segments were assigned to the different partitions representing the anatomic regions according to the radial degrees encompassed. Distance was assigned as the z-value for the line segments, with line features dissolved on region name. The data were subsequently displayed in ArcScene[9] using the base of each region as its base height and its z-value or distance from the dentate line as the extrusion factor. The 3D map of lesions was then overlaid and the entire map animated in ArcScene.

## Results

### 2D and 3D rendering and visualization

**Figure 3:**
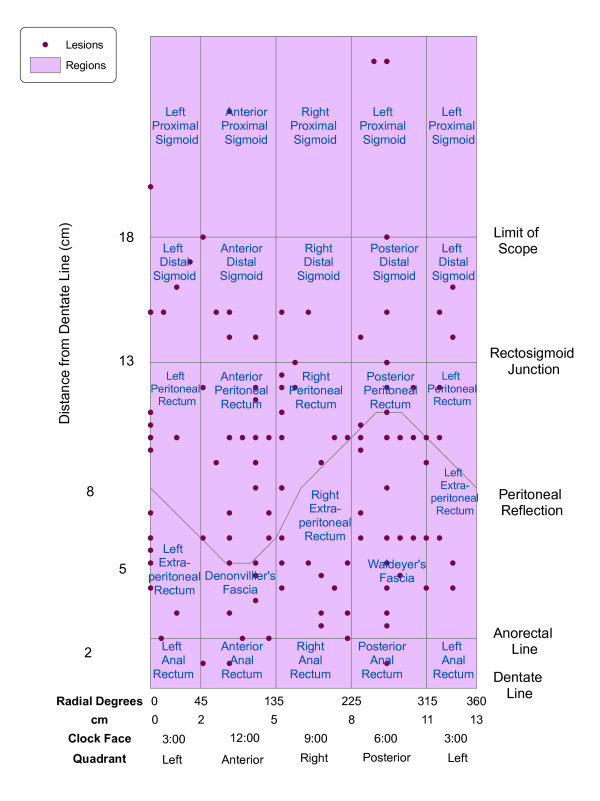
**TEM lesions overlaid on anatomic regions**. The results of 2D mapping are shown. The map of lesions (points) was overlaid on the map of anatomic regions (polygons). Note that regions on the left and right edges of the map represent a single split region as a result of the projection used.

Figure 3 demonstrates the results of the two-dimensional mapping process for anatomic regions and lesions. Region names reflect patient orientation (left/right, anterior/posterior) and proximity to fixed anatomic structures on the right vertical axis. Note that regions on the left and right edges of the map represent halves of the same region as a result of the projection used.

**Figure 4:**
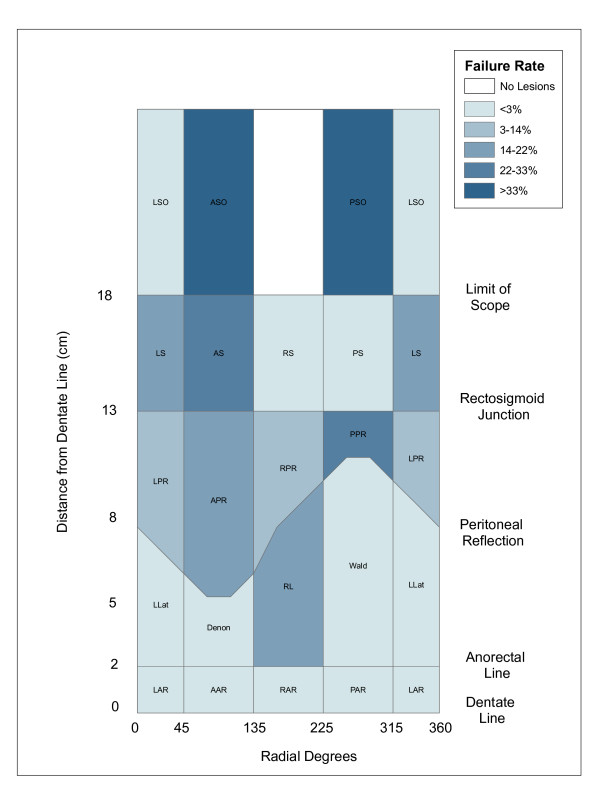
**Choropleth map of operative failure rates**. A choropleth map showing the geographic distribution of operative failure rates by anatomic region is shown. Darker colors indicate higher rates.

2D Choropleth mapping of procedural failure rates by anatomic region is shown in Figure 4. Visual inspection of this map revealed that higher rates of failure were associated with regions further from the dentate line, above the peritoneal reflection and above the rectosigmoid junction.

**Figure 5:**
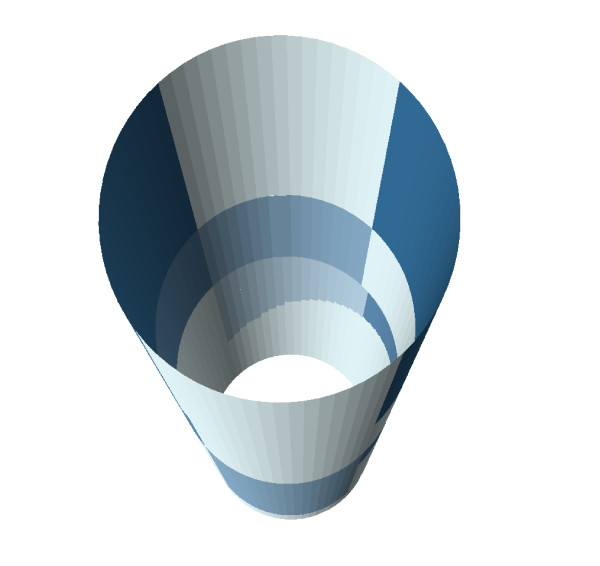
**3D choropleth map of operative failure rates**. A 3D version of the choropleth map shown in Figure 4. Dark-colored regions near the top of the figure represent regions of high failure rates at the greatest distance from the dentate line. Regions with highest rates are opposite one another, on the anterior and posterior aspects of the rectum.

Additional file 1 shows the results of the 3D mapping process for anatomic regions (polygons) and TEM lesions (points). The distortion (stretching) imposed by the 2D projection and the split representation of the border regions in 2D are corrected in 3D. Animation allows further exploration of the data from all perspectives. Transparent rendering [see Additional file 2] makes obvious that the peritoneal reflection is an oblique cross-sectional plane, as it exists in vivo. A 3D choropleth map of procedural failure rates (Figure 5) reveals an additional pattern not evident in 2D: anatomic regions with the highest rates were opposite one another in the anterior and posterior planes, with relative protection of the lateral regions.

### Statistical analysis

The lack of statistically significant global clustering across regions in operative failure rates could be due to the relatively small number of regions (20) resulting in low statistical power. As a result of 2D and 3D visualization, the two pathologic factors and four locational factors were chosen as independent variables in ordinary least squares regression on operative failure. These included average size of lesion, previous recurrence rate, distance from the dentate line, location of the region boundary on the rectal wall (in radial degrees) and location above the peritoneal reflection, above the rectosigmoid junction, or on the anterior/posterior (front/back) plane. While only distance from the dentate line was significant, both rectal wall location and location above the rectosigmoid junction were marginally significant (p < 0.08).

Mapping the residuals from the regression analysis demonstrated a pattern of greater residuals above the rectosigmoid junction (data not shown). This was verified by the finding of significant spatial clustering with Moran's I (p < 0.005). This indicated the presence of an unexplained spatial variation component of the linear regression model and that use of a spatial regression model to account for the spatial structure of the data was appropriate. Subsequent spatial regression analysis showed that only distance from the dentate line had a significant relationship to procedural failure. Failure rates were higher with increasing distance from the dentate line (p < 0.001).

## Discussion

The application of GIS to mapping anatomic features and clinical events has been infrequent in the GIS and medical literature. Among these, few have used GIS for anatomical mapping or feature attribute calculation. Our application is novel in several important ways. While the primary focus of other applications has been data integration for visualization of anatomy, the focus of this study was the spatial analysis of clinical patient data and the role of anatomy in clinical outcomes. No other investigators have used location or topology and spatial statistics for analyzing clinical events.

Based on empirical experience of a surgeon performing TEM, it appears that lesions at greater distance are more challenging to excise. However, it is not evident from clinical experience which specific locations that are distant from the anal verge are associated with procedural failure. The methodology as presented is the first to formally analyze location as a determinant of procedural failure. Rather than simply analyzing distance as a variable (i.e., near versus far), as has been studied in clinical studies using traditional statistical methodology, we demonstrate the importance of geographic features. The evidence from this paper shows that lesions on the anterior and posterior walls of the rectum that are distant are the most difficult to excise, which could be explained by the ability of the surgeon to properly position the patient or the scope.

The ability to visualize and analyze anatomic features and clinical events in a GIS is based on the existence of universal reference points for features. This allows data from multiple subjects with individual anatomic variability to be represented on a common anatomical map and analyzed in aggregate form. In the current study, all lesions were measured according to their distance from the dentate line (cm) and their clockface position (degrees). The dentate line and the upper limit of the scope (18 cm) formed the geographic extent of the map, while 90-degree cylindrical quadrants formed vertical boundaries of the anatomic regions. The location of the peritoneal reflection and the rectosigmoid junction, standard reference points in human anatomy textbooks, provided additional boundaries for the regions. Finally, the designations of near and far (proximal and distal) and left and right (an integral practice in describing the location of medical phenomena) completed our naming of the regions.

While imaging modalities such as CT and MRI may be able to detect rectal lesions, they do not provide the surgeon essential information regarding the location of polyps and lesions within the rectum in terms of distance from the anus, reference to clockface position, and relationships with the sphincter apparatus, prostate, vagina, and the peritoneal reflection. Utilizing an endoscopically-derived cylindrical coordinate map was necessary for providing clinically relevant data that could later guide surgeons in the performance of the rectal surgery, as current management relies on the determination of lesion position using rigid sigmoidoscopy, and not CT or MRI.

In the context of performance of TEM, the error introduced by utilizing a cylindrical model is tolerable since the surgical procedure is performed in a scope that is shaped like a cylinder. In addition, applying this towards most rectal surgery would be valid, since preoperative location is endoscopically determined utilizing rigid sigmoidoscopy, and since the rectum is a relatively fixed structure within the pelvis. However, applying this to flexible colonoscopy could potentially cause additional error into the system since the colonoscope is a mobile measuring device, and the colon is more flexible and redundant than the rectum.

Spatial referencing forms a cornerstone of traditional medical diagnosis and prognosis. For example, in describing the location of cancerous lesions of the breast, quadrant and proximity to the nipple are important designations. Depth of invasion and metastasis to lymph nodes or other tissues carries prognostic significance for all types of cancer. Lobe (right or left) and proximity to common surface landmarks such as the umbilical fissure are used in describing the location of liver disease. Abdominal aortic aneurysms are described in part by their relationship with vascular landmarks such as the aortic bifurcation and the kidneys.

The usefulness of spatial referencing of anatomic regions for this study was validated by the finding of significant global spatial variation in the linear regression residuals, indicating unexplained spatial variation in the original linear regression. In addition, the finding that locational variables were the only ones to significantly affect operative failure indicated that spatial location is important in this context.

This standardization of anatomic landmarks within the human body becomes the key for establishing spatial reference points for GIS registration and allows extension of the current work to the mapping of other organs or anatomic structures. Visualization of human anatomic features is not a new or revolutionary concept. More realistic and detailed visual models exist in all varieties of media and date back to the first textbook of anatomy. The unique contribution of the current work is the establishment of a GIS base layer of anatomic regions on which to overlay the location of clinical events so that spatial attributes such as distance, topology and depth can be analyzed and modeled. Clustering and other spatial patterns can be identified and quantified, and the relationship of spatial location to clinical outcomes can be modeled through multivariate analysis. Moreover, 3D visualization and animation allows exploration of the data from all perspectives, eliminating the distortion inherent in 2D projection. Consequently, spatial relationships are more accurately represented, with visualization of patterns not evident in 2D. Appearance of features begins to resemble clinical reality and therefore easier for the clinician to interpret. Transparent rendering can further enhance visualization of the regions, as is the case of the oblique cross-sectional plane of the peritoneal reflection which is only apparent when transparency is increased.

The process described here adds a new dimension to clinical prognosis and assessment of outcomes which has until recently gone untapped.

## Conclusions and future study

This study has demonstrated that spatial rendering of anatomic and pathologic features is feasible in a GIS. This process is innovative to the methodology of medical outcomes research in that it allows the ability to study the relationship of anatomic position to clinical events. The relative effects of multiple independent variables and interactions among them may be further explored through the use of multivariate spatial analysis.

The visualization and spatial analysis of clinical data increases our awareness of the relationship between anatomic features and disease outcomes. This may lead to new and greater understanding of the disease process and therapeutic interventions which can be translated into better clinical practices and improved patient outcomes. Furthermore, increased awareness of the limitations of a procedure like TEM will further guide the proper indications for its use.

## Competing interests

The author(s) declare that they have no competing interests.

## Authors' contributions

JG performed all database and GIS functions and developed the sequence for rendering the data in the GIS. She also participated in the study design, performed all statistical analyses, and drafted and revised the manuscript. SG conceived of the study, participated in the study design, collected the data, contributed the mathematical formulas for transforming TEM measurements into x-y and x-y-z data, and helped draft and revise the manuscript. CB contributed to the methodology for rendering the data in 3D. RBW and RS participated in the study design and helped draft the manuscript. All authors read and approved the final manuscript.

## Appendix I: The TEM mapping process

Mapping was done with ArcGIS 9.1 (ArcGIS menu choices are given in bold text).

A. Plotting lesions: 2D (refer to Table 1)

**Table 1:** Sample data points for lesions (partial)

	*2D Coordinates*			*Lesion Attributes*				*3D Coordinates*		
**Lesion ID**	**θ **degrees	**y **distance	**x**	**Size**	**Conversion**	**Breech**	**OR Time **(Mins)	**x**_**1**_	**y**_**1**_	**z**

1	270	10	9.425	2.2	0	0	325	0.000	-1.000	-10
2	270	11	9.425	3	1	0	150	0.000	-1.000	-11
3	120	10	4.189	4	0	1	80	-0.500	0.866	-10
4	135	6	4.712	4.7	1	0	135	-0.707	0.707	-6
5	90		3.142	4	0	0	50	0.000	1.000	0
6	270	13	9.425	1	0	0	51	0.000	-1.000	-13
7	285	10	9.948	3.5	0	0	120	0.259	-0.966	-10
8	165	13	5.760	3.3	1	0	110	-0.966	0.259	-13
9	0	15	0.000	2	0	1	120	1.000	0.000	-15
10	285	4.5	9.948	3.5	0	0	160	0.259	-0.966	-4.5
11	135	7	4.712	4	0	0	120	-0.707	0.707	-7
12	300	12	10.472	2.4	1	0	135	0.500	-0.866	-12
13	300	10	10.472	2.5	0	0	130	0.500	-0.866	-10
14	90	7	3.142	5.5	0	0	240	0.000	1.000	-7
15	240	10	8.378	2.5	1	0	120	-0.500	-0.866	-10
16	120	5	4.189	2.5	0	0	114	-0.500	0.866	-5
17	270	5	9.425	1	0	1	50	0.000	-1.000	-5
18	0	10	0.000	2.5	0	0	20	1.000	0.000	-10

1. Create a table in D-Base, Access or comma delimited (See Table 1) and enter values for lesion locations as clock face position (θ) and distance (y) from dentate line.

2. Convert degrees (θ) to x-coordinates in centimeters:

x = (θ/360)*4π, where 4π = circumference of the scope in centimeters (diameter = 4 cm)

3. Add data table to map in ArcMap (**File-Add Data**).

4. Plot x, y coordinates (**Tools-Add XY Data**).

B. Plotting anatomic regions: 2D

5. Create a second table (see Table 2) to hold the region boundary coordinates.

**Table 2:** 2D and 3D region coordinates (partial)

**Region name**	**θ **degrees	**y **distance	**x**	**r **radians	**x**_**1**_	**y**_**1**_	**z**
L. Anterior Rectum	0	0.000	0.000	0.000	1.000	0.000	0.000
Anterior Anal Rectum	45	0.000	1.571	0.785	0.707	0.707	0.000
R. Anal Rectum	135	0.000	4.712	2.356	-0.707	0.707	0.000
Posterior Anal Rectum	225	0.000	7.854	3.927	-0.707	-0.707	0.000
L. Anterior Rectum	315	0.000	10.996	5.498	0.707	-0.707	0.000
L. Anterior Rectum	360	0.000	12.566	6.283	1.000	-2.45E-16	0.000
L. Lateral	0	2.000	0.000	0.000	1.000	0.000	-2.000
Denonvilliers	45	2.000	1.571	0.785	0.707	0.707	-2.000
R. Lateral	135	2.000	4.712	2.356	-0.707	0.707	-2.000
Waldeyers	225	2.000	7.854	3.927	-0.707	-0.707	-2.000
L. Lateral	315	2.000	10.996	5.498	0.707	-0.707	-2.000
L. Lateral	360	2.000	12.566	6.283	1.000	-2.45E-16	-2.000
*L. Peritoneal Rectum*	*0*	*8.000*	*0.000*	*0.000*	*1.000*	*0.000*	*-8.000*
*L. Peritoneal Rectum*	*5*	*7.739*	*0.175*	*0.087*	*0.996*	*0.087*	*-7.739*
*L. Peritoneal Rectum*	*10*	*7.479*	*0.349*	*0.175*	*0.985*	*0.174*	*-7.479*
*L. Peritoneal Rectum*	*15*	*7.224*	*0.524*	*0.262*	*0.966*	*0.259*	*-7.224*
*L. Peritoneal Rectum*	*20*	*6.974*	*0.698*	*0.349*	*0.940*	*0.342*	*-6.974*
*L. Peritoneal Rectum*	*25*	*6.732*	*0.873*	*0.436*	*0.906*	*0.423*	*-6.732*
*L. Peritoneal Rectum*	*30*	*6.500*	*1.047*	*0.524*	*0.866*	*0.500*	*-6.500*
*L. Peritoneal Rectum*	*35*	*6.279*	*1.222*	*0.611*	*0.819*	*0.574*	*-6.279*
*L. Peritoneal Rectum*	*40*	*6.072*	*1.396*	*0.698*	*0.766*	*0.643*	*-6.072*
*L. Peritoneal Rectum*	*45*	*5.879*	*1.571*	*0.785*	*0.707*	*0.707*	*-5.879*
**...**	**...**	...	**...**		...	...	...
L. Peritoneal Rectum	360	8.000	12.566	6.283	1.000	0.000	-8.000
**...**	**...**	...	**...**	**...**	...	...	...
Top boundary	0	26.000	0.000	0.000	1.000	0.000	-26.000
Top boundary	45	26.000	1.571	0.785	0.707	0.707	-26.000
Top boundary	135	26.000	4.712	2.356	-0.707	0.707	-26.000
Top boundary	225	26.000	7.854	3.927	-0.707	-0.707	-26.000
Top boundary	315	26.000	10.996	5.498	0.707	-0.707	-26.000
Top boundary	360	26.000	12.566	6.283	1.000	0.000	-26.000

6. Enter coordinates of region boundaries as previously described in text (See **Two-dimensional (2D) rendering**).

7. Calculate the coordinates of the peritoneal reflection:

a. x = θ, where θ = radial degrees from 0 to 360

b. y = 8 - 3sinθ

c. add x, y for every 5 degrees from 0 to 360.

8. Add table to map (**File-Add Data**) and plot the x, y coordinates (**Tools-Add XY Data**).

9. Create a new polygon layer to contain anatomic regions (**ArcToolbox-Data Management Tools-Feature class-Create Feature Class**).

10. Digitize regions from points plotted in step 8. Use snapping (**Editor-Snapping**) to aid in selecting points to define vertices of region.

11. Add attribute data, such as region name, to polygons (**Open Attribute Table-Add Field**).

C. Plotting anatomic regions: 3D (refer to Table 2)

1. Convert θ (degrees) to radians (rad)

2. Convert region cylindrical coordinates (θ, d) to Cartesian coordinates (x, y, z) in centimeters, assuming a diameter of the rectum of 4 cm

a. x_1 _= 2cos(rad)

b. y_1 _= 2sin(rad)

c. z = -d

3. For every region, add points every 5 degrees between the bounding coordinates to create a more cylindrical shape.

4. Add data table to map in *ArcMap *(**File-Add Data**).

5. Plot x, y coordinates (**Tools-Add XY Data**).

6. Add an ID field to the attribute table of the point feature class. (**Open Attribute Table-Add Field**). This allows easier sequential creation of lines in next step. Label each point with the ID of the anatomic region to which it belongs.

7. Create new line feature class (**ArcToolbox-Data Management Tools-Feature class-Create Feature Class**). (Refer to Table 3)

**Table 3:** 3D Line feature Class (partial)

**OBJECTID**	**Region name**	**z**_**1**_	**z**_**2**_	**Base**	**z**
1	L. Anal Rectum	2.000	2.000	0.000	2.000
2	Ant. Anal Rectum	2.000	2.000	0.000	2.000
3	R. Anal Rectum	2.000	2.000	0.000	2.000
4	Post. Anal Rectum	2.000	2.000	0.000	2.000
5	L. Anal Rectum	2.000	2.000	0.000	2.000
6	L. Lateral	8.000	7.739	2.000	8.000
7	L. Lateral	7.739	7.479	2.000	7.609
8	L. Lateral	7.479	7.224	2.000	7.351
9	L. Lateral	7.224	6.974	2.000	7.099
10	L. Lateral	6.732	6.500	2.000	6.616
11	L. Lateral	6.500	6.279	2.000	6.390
12	L. Lateral	6.279	6.072	2.000	6.176
13	L. Lateral	6.072	5.879	2.000	5.975
14	Denonvilliers	5.879	5.702	2.000	5.790
15	Denonvilliers	5.702	5.543	2.000	5.622
16	Denonvilliers	5.543	5.402	2.000	5.472
17	Denonvilliers	5.402	5.281	2.000	5.342
18	Denonvilliers	5.281	5.181	2.000	5.231
19	Denonvilliers	5.181	5.102	2.000	5.142
20	Denonvilliers	5.102	5.046	2.000	5.074
21	Denonvilliers	5.046	5.011	2.000	5.028
22	Denonvilliers	5.011	5.000	2.000	5.006
23	Denonvilliers	5.000	5.011	2.000	5.006
24	Denonvilliers	5.011	5.046	2.000	5.028
25	Denonvilliers	5.046	5.102	2.000	5.074
26	Denonvilliers	5.102	5.181	2.000	5.142
27	Denonvilliers	5.181	5.281	2.000	5.231
28	Denonvilliers	5.281	5.402	2.000	5.342
29	Denonvilliers	5.402	5.543	2.000	5.472
30	Denonvilliers	5.543	5.702	2.000	5.622
31	Denonvilliers	5.702	5.879	2.000	5.790

a. Create a series of line features by connecting sequential points in point feature class created in Step 1. Use snapping (**Editor-Snapping**) to aid in selecting points to define vertices of region line.

b. Obtain z-values obtained from rectal topology sketch (Figure 1) and add these to the line feature class (**Open Attribute Table-Add Field**))

*For regions bounded by rectosigmoid junction*,

c. Z-value = midpoint of Z1 and Z2 (difference in value of Z1 and Z2 added to smaller z-value, where

Z1 = z-value of beginning node in line

Z2 = z-value of end node in line

Values for Z1 and Z2 are copied from points file created in step 1 using Attribute Transfer Tool. (**Spatial Adjustment-Attribute Transfer Mapping**).

d. Add Base Height field as attribute (**Open Attribute Table-Add Field**)

Base for regions are obtained from rectal topology sketch (Figure 1)

For regions with Base = rectosigmoid junction, Base = Z-value of bounding region below

8. Display regions in *ArcScene *by extruding the line data. Extrusion turns lines into walls. Add line data to *ArcScene *scene (**File-Add Data**) and set layer properties:

a. set base height expression = **[Base]**

b. set extrusion expression = **[Z] - [Base]**

c. Apply extrusion by: **adding to each feature's base height**

D. Plotting lesions: 3D

1. In *ArcMap*, using Table 1 (right columns), convert θ to x, y coordinates in centimeters, assuming a diameter of the rectum of 4 cm

a. x1 = 2cosθ

b. y1 = 2sinθ

c. z = - distance field

2. Add data table to map (**File-Add Data**).

3. Plot x_1_, y_1 _coordinates (**Tools-Add XY Data**) and export data to save coordinates (**right-click event layer- Export data**).

4. Display in *ArcScene*

a. set base heights to [z] – points will float in space

b. Overlay on Regions layer

## Supplementary Material

Additional File 1 Animated 3-D rendering of anatomic regions with lesions. Animated 3-dimensional visualization of features allows exploration from all perspectives. Click here for file

Additional File 2 Animated transparent 3-D anatomic regions. Transparent rendering in ArcScene allows features to be highlighted more clearly. The peritoneal reflection appears as an oblique plane in cross-section. Click here for file
